# Factors associated with severe pneumonia among children <5 years, Kasese District, Uganda: a case-control study, January–April 2023

**DOI:** 10.1186/s41479-024-00134-y

**Published:** 2024-07-25

**Authors:** Mercy Wendy Wanyana, Richard Migisha, Patrick King, Abraham Kibaba Muhesi, Benon Kwesiga, Daniel Kadobera, Lilian Bulage, Alex Riolexus Ario

**Affiliations:** 1 Uganda Public Health Fellowship Program, Uganda National Institute of Public Health, Kampala, Uganda; 2 Kasese District Local Government, Kasese, Uganda; 3 Ministry of Health, Kampala, Uganda

**Keywords:** Severe pneumonia, Children < 5 years, Uganda

## Abstract

**Background:**

Pneumonia is one of the leading causes of infant mortality globally, particularly in sub-Saharan Africa. In Uganda, pneumonia was the fourth leading cause of death in children <5 years in 2018. Analysis of 2013–2022 data for children <5 years from the District Health Information System indicated a high incidence of severe pneumonia in Kasese District, Uganda. We investigated to identify factors associated with severe pneumonia among children <5 years in Kasese District to inform prevention and control strategies.

**Methods:**

We conducted a 1:1 hospital-based case-control study among children aged 2–59 months presenting with pneumonia at five high-volume facilities in Kasese District from January to April 2023. A case was defined as pneumonia with ≥1 of the following danger signs: low oxygen saturation, central cyanosis, severe respiratory distress, feeding difficulties, altered consciousness, and convulsions. Controls were outpatient children aged 2–59 months with a diagnosis of non-severe pneumonia. We reviewed medical records at facilities and used an interviewer-administered questionnaire with caregivers to obtain information on socio-demographic and clinical characteristics. Logistic regression was used to identify factors associated with severe pneumonia.

**Results:**

We enrolled 199 cases and 174 controls. The odds of severe pneumonia were higher among children with diarrhoea only (adjusted odds ratio [aOR] = 2.9, 95%CI: 1.7–4.9), or malaria and diarrhoea (aOR = 3.4, 95%CI: 2.0-5.9), than those without a co-existing illness at the time of pneumonia diagnosis. Not being exclusively breastfed for ≥ 6 months (aOR = 2.0, 95%CI: 1.1–3.3) and exposure to indoor air pollution from cooking combustion sources (aOR = 2.9, 95%CI: 1.8–4.7) increased odds of severe pneumonia.

**Conclusion:**

The findings highlight the significance of comorbidities, lack of exclusive breastfeeding, and exposure to indoor air pollution in the development of severe pneumonia. Promoting exclusive breastfeeding for ≥ 6 months and advocating for the use of clean energy sources, could mitigate morbidity attributable to severe pneumonia in the region.

## Background

Pneumonia, a largely preventable disease, continues to pose a significant public health challenge for children <5 years. In 2019, it emerged as the primary infectious cause of death in this age group, representing 14% of total fatalities globally [1]. Notably, half of all pneumonia cases and related deaths occur in sub-Saharan Africa [2].

Pneumonia is categorized into non-severe and severe based on clinical severity and risk of adverse outcomes. Severe pneumonia, which often necessitates hospitalisation, carries a higher case fatality rate and is associated with long-term morbidity and potential lifelong disability [3, 4]. Between 2015 and 2019, McAllister et al., documented a worrying trend of severe pneumonia among children <5 years in sub-Saharan Africa with a seven-fold increase in severe pneumonia cases [2]. This suggested a growing burden of severe pneumonia in this region.

In Uganda, the incidence of severe pneumonia was 108 per 100,000 children under five years in 2022 based on the routinely collected weekly national surveillance data [5]. Furthermore, analysis of trends and spatial distribution of pneumonia hospital admissions and mortality among children <5 years in Uganda in the period 2013–2021, indicated a particularly high incidence of pneumonia admissions in Kasese District [6]. During this period, the incidence of pneumonia admissions was >3000 per 100,000 children <5 years in all years with the highest of 7,421 cases per 100,000 in 2019. This growing burden creates a need to understand the factors associated with severe pneumonia among children <5 years in order to implement effective evidence-based control and prevention strategies. A previous study by Rudan et al., found that about 7–13% of pneumonia cases progress to the severe form; however, the risk factors for progression to severe pneumonia are not well documented in Uganda [7]. In low and middle-income countries, documented risk factors for severe pneumonia encompass bacterial causes, immunization status, low weight-for-age, limited caregiver education, lower wealth quintile, delayed care-seeking, household overcrowding, co-morbidities, malnutrition, and young age [8–10]. However, these factors exhibited inconsistencies and variations, influenced by unique contextual disparities across different settings. Limited documentation exists on factors linked to severe pneumonia development among children under five in Uganda, particularly in high-burden areas like Kasese District. Our study aimed to determine factors associated with severe pneumonia development among children under five in Kasese District, to inform tailored control and prevention strategies.

## Methods

### Study setting

**Fig. 1:**
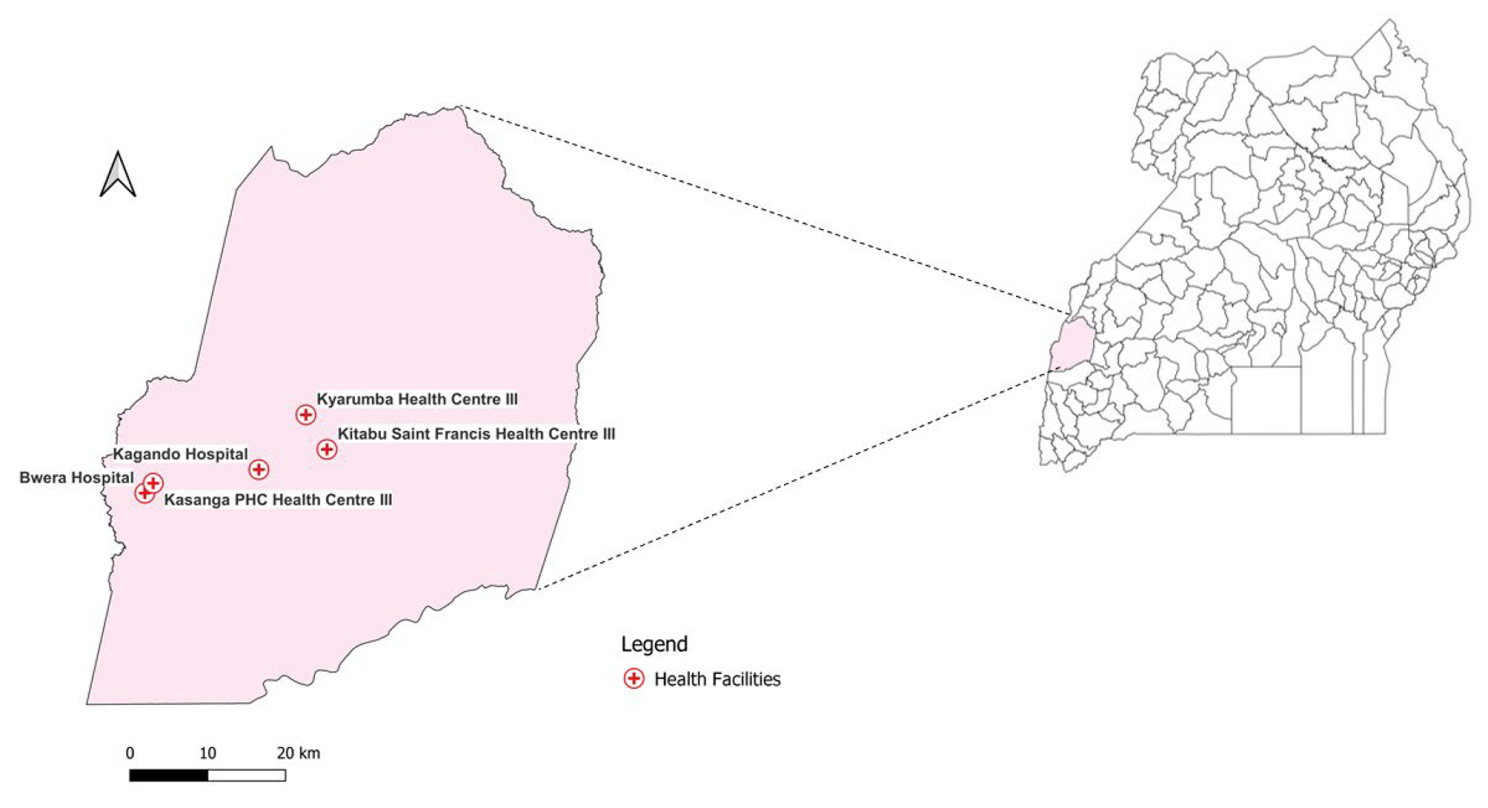
Health facilities where study was conducted in Kasese District, Uganda, January to April 2023

We conducted the study in Kasese District located in Western Uganda. Kasese is one of the districts where the Integrated Community Case Management for Childhood Illnesses including diarrhoea, malaria, and pneumonia were being implemented [11]. There were 139 health facilities in Kasese District, 81 health centre IIs, 48 health centre IIIs, 6 health centre IVs and 4 general hospitals. As per the revised package of basic health services for Uganda, 2014, severe pneumonia can only be managed at health centre IIIs, health centre IV, and general hospitals [12]. We specifically conducted the study in five health facilities reporting the highest number of severe pneumonia cases. These included Kagando Hospital, Bwera Hospital, Kasanga PHC Health Centre III, Kyarumba HC III, and St Francis of Assis-Kitabu Health Centre III (Fig. 1). These facilities offer both in-patient and outpatient childcare services.

### Study design

We conducted an unmatched hospital-based case-control study between January to April 2023. We defined severe pneumonia cases as presentation with pneumonia with any of the following danger signs: oxygen saturation <90%, central cyanosis, severe respiratory distress, inability to drink or breastfeed or vomiting everything, altered consciousness, and convulsions in a child aged 2–59 months [13] attending five high-volume facilities in Kasese District from January to April 2023. A control was a child aged 2–59 months presenting with lower chest wall indrawing or fast breathing (respiratory rate ≥ 50 breaths per min if aged 2–11 months; ≥40 breaths per min if aged 12–59 months) and without signs of severe pneumonia at the respective health facilities during the same period as cases.

We determined the sample size using the Fleiss et al. formula [14] using Open Epi software. We assumed a two-sided confidence level (CI) = 95%, power = 80%, 1:1 ratio of cases to controls, taking non-exclusive breastfeeding as a main predictor for severe pneumonia with an odds ratio of 2.7 [15], and percentage of exposed controls at 9% [16]. We obtained a total sample of 153 cases and 153 controls. Controls were selected from those registered after a case at the same facility. We reviewed medical records at facilities and used an interviewer-administered questionnaire with caregivers in their homes to obtain information on clinical and non-clinical characteristics.

Using a structured questionnaire, we collected data regarding socio-demographics, child’s health, health-seeking behaviour, and environmental-related factors from both the cases and controls. We further reviewed medical records and child health cards to obtain more details on the clinical characteristics including the immunization status of the children.

Socio-demographic factors included age, sex, birth order, caregiver’s education level, and wealth status. We derived wealth status as a composite variable using principal component analysis of data on ownership of consumer items and livestock, characteristics of the dwelling unit, water sources and sanitation facilities.

Child health factors included nutritional status categorised into normal stunting, wasting, underweight and overweight. This was assessed by the following MUAC (in cm), weight for age Z score (WAZ), weight for height/length Z score (WHZ), and height for age Z score (HAZ). Breastfeeding status was categorised into exclusively breastfed for ≥ 6 months and those who were not as defined by WHO classification of breastfeeding [17]. Underlying illness was defined as having diarrhoea, measles, malaria or HIV during the current pneumonia illness. Immunisation status was defined as age-appropriate receipt of vaccines (including Pneumococcal Conjugate Vaccine (PCV), DPT-HebB + Hib2, measles and polio vaccines) as indicated in the Uganda Immunisation schedule [18].

Environmental factors included type of cooking fuel used at home, exposure to household air pollution from cooking fuel assessed using proximity to cooking combustion sources during home cooking with the child in the kitchen and exposure to tobacco smoke defined as staying with a cigarette smoker in the same household. Health-seeking behaviour factors included time to seeking care following caregiver’s recognition of illness, type of healthcare sought for the episode of illness prior to enrolment in the study and place where healthcare was sought for this episode of illness prior to enrolment in the study: home remedies, village health team, clinic, drug shop, health centre II, health centre III, and health centre IV.

### Data analysis

We downloaded data in an Excel file and imported in STATA 16 software (StataCorp, Texas USA) for analysis. We tabulated categorical variables as frequencies with corresponding percentages. For continuous variables with a skewed distribution, such as the time to care-seeking following the caregiver’s recognition of illness, we reported medians along with their interquartile ranges (IQR). We used logistic regression to obtain independent variables significantly associated with having severe pneumonia. The independent variables with p-values ≤ 0.2 at bi-variate analysis were used to develop a multivariable logistic regression model using a forward stepwise approach. The strength of association between outcome variable and the independent variables of interest were assessed by calculating odds ratios (ORs) with 95% confidence intervals. Variables with *p* < 0.05 were considered statistically significant.

## Results

### Enrolment of participants

**Fig. 2:**
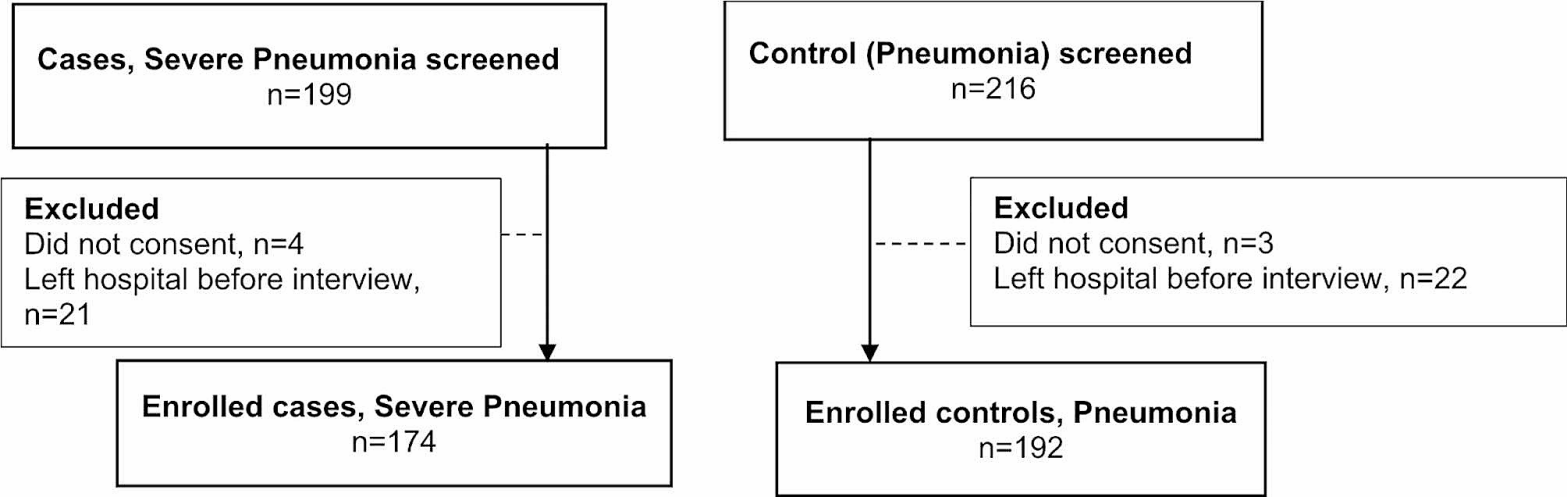
Flow diagram of enrolment of participants and reasons for exclusion

The study included 366 children <5 years, 174 cases and 192 controls (Fig. 2).

### Socio-demographic characteristics of study participants

The median age of children was 12 months (IQR: 6–24); 60% were males and 44% were in the ≤ 11 months age group. Nearly half (48%) of children were in the 1–2 birth order with 62% with primary caregivers whose highest level of education was primary education and 21% from households in the highest wealth quintile. Slightly more controls were in 1–2 birth order category (54% vs. 41%) and female (44% vs. 36%) (Table 1).

**Table 1:** Socio-demographic characteristics of study participants, Kasese District, Uganda, January to April 2023

Characteristic	Cases		Controls		*P*-value
	*n*	(%)	*n*	(%)	
**Sex**					0.092
Male Female	112 62	(64) (36)	107 85	(56) (44)	
**Age group**					0.489
≤ 11 months 12–23 months ≥ 24months	78 47 49	(45) (27) (28)	84 44 64	(44) (23) (33)	
**Birth order**					0.063
1– 2 3–4 ≥ 5	72 60 42	(41) (35) (24)	103 53 36	(54) (28) (19)	
**Primary caregiver’s level of education**					0.689
None Primary level Second Tertiary	22 113 36 3	(12) (65) (21) (2)	26 115 45 6	(14) (60) (23) (3)	
**Wealth Status**					0.248
Upper tertile Middle tertile Low tertile	52 59 63	(30) (34) (36)	73 60 59	(38) (31) (31)	

### Factors associated with severe pneumonia among children <5 years in a hospital-based case-control study assessing factors associated with severe pneumonia in Kasese District, Uganda

At multivariable analysis, children who were not exclusively breastfed had twice the odds (aOR:1.97,95% CI:1.13–3.36) of having severe pneumonia compared to children who were exclusively breastfed for ≥6 months (Table 2). Children with diarrhoea only had approximately 3 times the odds (aOR:2.88,95% CI:1.69–4.89) of having severe pneumonia compared to children with no underlying disease at the time of their pneumonia-related diagnosis. Children with malaria and diarrhoea had 3 times the odds (aOR:3.41,95% CI:1.98–5.86) of having severe pneumonia compared to children with no underlying disease at the time of their pneumonia-related diagnosis (Table 2). Children who were exposed to indoor air pollution through proximity to cooking combustion sources during home cooking with the child in the kitchen had 3 times higher odds of developing severe pneumonia (aOR:2.89,95% CI:1.77–4.73) (Table 2).

**Table 2:** Factors associated with severe pneumonia among children <5 years in Kasese District, Uganda, January to April 2023

Characteristic	Case	Control	Unadjusted analysis			Adjusted analysis		
	*n*	*n*	cOR	(95% CI)	*p*-value	aOR	(95% CI)	*p*-value
**Birth order**								
1–2 3–4 ≥ 5	72 60 42	103 53 36	Ref 1.62 1.67	(1.01–2.61) (0.98–2.86)	0.047* 0.062	1.55 1.40	(0.82–2.93) (0.72–2.70)	0.176 0.331
**Wealth Status**								
Upper tercile Middle tercile Low tercile	52 59 63	73 60 59	Ref 1.37 1.74	(0.83–2.27) (1.05-0.90)	0.212 0.032*	1.05 1.51	(0.56–1.96) (0.82–2.78)	0.185 0.889
**Exclusive breastfeeding for ≥6 months**								
Yes No	50 124	75 117	Ref 1.80	(1.13–2.87)	0.013*	1.97	(1.13–3.46)	0.017*
**Underlying illness**								
None Malaria only Diarrhoea only Malaria and diarrhoea	65 13 52 44	108 4 37 33	Ref 0.85 2.542.76	(0.49–1.48) (1.63–3.93) (1.69–4.56)	0.575 < 0.001* < 0.001*	0.99 2.88 3.41	(0.88–1.12) (1.69–4.89) (1.98–5.86)	0.937 < 0.001* < 0.001*
**Place of first care**								
Health Facility VHTs Home remedies	70 65 27	55 102 22	Ref 0.50 0.96	(0.31–0.80) (0.50–1.87)	0.004* 0.915	0.78 1.33	(0.44–1.39) (0.63–2.80)	0.404 0.449
**Exposure to indoor air pollution through proximity to cooking combustion sources during home cooking with child in the kitchen**								
Yes No	98 54	79 76	1.89 Ref	(1.19–2.98)	0.007*	2.89	(1.77–4.72)	< 0.0001*

**aOR**: Adjusted odds ratio; **CI**: Confidence interval; **cOR**: Crude odds ratio; **VHT**: Village health team

## Discussion

We assessed factors associated with severe pneumonia among children <5 years in five high volume health facilities in Kasese District. Severe pneumonia was found to be associated with factors such as non-exclusive breastfeeding, the presence of diarrhoea alone, and the co-occurrence of diarrhoea and malaria at the time of pneumonia diagnosis. Additionally, exposure to indoor air pollution, particularly in proximity to cooking combustion sources during home cooking with the child present in the kitchen, was identified as another significant contributing factor to the severity of pneumonia in children.

Children who were not exclusively breastfed for ≥6 months had twice the odds of developing severe pneumonia compared to those who were exclusively breastfed. This is consistent with a systematic review of studies largely outside the African Region done by Lamberti et al. that found that children who were not exclusively breastfed had a 5-fold increased risk of developing severe pneumonia which often requires hospitalisation [19]. Breast milk contains antibodies which enhance the child’s immune system and strengthen defence mechanisms against infectious agents protecting the child from developing severe disease [14, 15]. Our findings highlight the need for exclusive breastfeeding to reduce the risk of developing severe pneumonia.

Children with comorbid diarrhoea or malaria and diarrhoea had higher odds of getting severe pneumonia compared to children with no underlying illness. Previous studies have indicated that malaria predisposes children to bacterial infections of common pneumonia-causing organisms such as *Streptococcus Pneumoniae* and *Klebsiella Pneumoniae* often with severe forms of the disease [20]. Similarly, diarrhoea may predispose children to pneumonia through increases in loss of zinc in diarrhoea stools, electrolyte imbalances and renal losses of vitamin A [21]. Due to their immature immune system, children already suffering from an illness are more likely to suffer from severe illness if they get another coinfection [22]. Strengthening integrated management of childhood illnesses which combines prevention and case management of the three illnesses while providing a holistic approach to child health is therefore critical in preventing severe pneumonia [23].

Children who were exposed to indoor air pollution through proximity to cooking combustion sources during home cooking with child in the kitchen had twice the odds of developing severe pneumonia compared to those who were not. In the study setting, like other parts of Africa, solid fuels such as firewood and charcoal are the main source of energy [24]. Combustion of wood during cooking releases air pollutants such as PM_2.5_ [25]. A previous study conducted in Ugandan kitchens where charcoal and firewood were used indicated PM_2.5_ concentrations higher than the World Health Organization 24-h Air Quality Guidelines [26]. Under these circumstances, children whose caregivers cook with them often get exposure to these air pollutants. Such exposure compromises their immune response against invading pathogens in the respiratory tract which predisposes them to more severe disease [27]. Use of clean energy sources such as electricity, biogas and natural gas may reduce the risk of developing severe pneumonia in this setting [28].

Overall, our findings point towards the need for integrated management of childhood illness by demonstrating significance of other child health factors namely: multimorbidity, lack of exclusive breastfeeding, and exposure to indoor air pollution, in the development of severe pneumonia. They further highlight the need for improving community and family practices such as exclusive breastfeeding and exposure to indoor air pollution from cooking combustion sources in preventing severe pneumonia. Given the contribution of multi-morbidities in the development of severe pneumonia in the current study, strengthening integrated management of childhood illnesses as a holistic approach to child health could potentially reduce the burden severe pneumonia.

Our study has some limitations. To ensure case ascertainment, we selected hospital-based controls. Hospital-based pneumonia cases maybe different from community controls [29]. Due to the differences in the diagnostic capacities of the selected health facilities, we did not explore clinical features and causative microorganisms associated with severe pneumonia. This might have resulted in residual confounding.

## Conclusion

Having an underlying illness, not being exclusively breastfed for ≥6 months, and exposure to indoor air pollution through proximity to cooking combustion sources during home cooking with the child in the kitchen were associated with developing severe pneumonia. Promoting exclusive breastfeeding for ≥6 months and advocating for the use of clean energy sources, such as clean energy stoves and integrated management of childhood illnesses at community and health facility level could reduce the burden of severe pneumonia in children <5 years in the region.

## Data Availability

No datasets were generated or analysed during the current study.

## Abbreviations

aOR: Adjusted Odds Ratio
cOR: Crude Odds Ratio
HAZ: Height-for-Age Z-score
IQR: Interquartile Range
MUAC: Middle Upper Arm Circumference
OR: Odds Ratio
VHT: Village Health Team
WAZ: Weight-for-Age Z-score
WHZ: Weight-for-Height/length Z-score
